# Sour Cherry Pomace Valorization as a Bakery Fruit Filling: Chemical Composition, Bioactivity, Quality and Sensory Properties

**DOI:** 10.3390/antiox12061234

**Published:** 2023-06-07

**Authors:** Nemanja Teslić, Jovana Kojić, Branislava Đermanović, Ljubiša Šarić, Nikola Maravić, Mladenka Pestorić, Bojana Šarić

**Affiliations:** Institute of Food Technology, University of Novi Sad, Bulevar cara Lazara 1, 21000 Novi Sad, Serbia; jovana.kojic@fins.uns.ac.rs (J.K.); branislava.djermanovic@fins.uns.ac.rs (B.Đ.); ljubisa.saric@fins.uns.ac.rs (L.Š.); nikola.maravic@fins.uns.ac.rs (N.M.); mladenka.pestoric@fins.uns.ac.rs (M.P.); bojana.saric@fins.uns.ac.rs (B.Š.)

**Keywords:** sour cherry pomace, semi-industrial scale, holistic approach, circular economy, food safety, proximate analysis, rheological properties, thermal stability, consumer test

## Abstract

Sour cherry pomace filling (SCPF) and commercial sour cherry filling (CSCF) produced on a semi-industrial scale were tested and compared in terms of food safety, chemical composition, bioactivity, quality, sensory properties and thermal stability. Both samples were safe for human consumption, thermally stable and there was a lack of syneresis. SCPF had a significantly higher fiber concentration (3.79 g/100 g) due to higher skin fraction and is considered a “source of fibers”. The higher skin fraction in SCPF also resulted in a higher mineral quantity (Fe—3.83 mg/kg fw) in comparison to CSCF (Fe—2.87 mg/kg fw). Anthocyanins concentration was lower in SCPF (7.58 mg CGE/100 g fw), suggesting that a significant amount of anthocyanins was removed from SC skin during juice extraction. However, there was a lack of statistical differences in antioxidant activity between the two fillings. CSCF was more spreadable, not as firm and less sticky, with lower storage and loss modulus values than SCPF. However, both fillings exhibited acceptable rheological and textural behaviour for fruit fillings. According to the consumer pastry test, 28 participants preferred each pastry; thus, there was a lack of preference toward any of the tested samples. SCP could be used as a raw material for the bakery fruit fillings industry, which leads to the valorization of food industry by-products.

## 1. Introduction

World production of sour cherry (*Prunus cerasus* L.), also called tart cherry, has generally increased in the period between 2006 (1,142,366 Tn) and 2021 (1,514,665 Tn) [1]. Compared to sweet cherries, many sour cherry varieties have a significantly lower sugar/acid ratio, which can make them excessively acidic for direct consumption in their fresh form. Therefore, sour cherries are generally considered an industrial fruit, and most of the fruit production is processed by the juice, jam or topping industries [2]. Among these industries, juice production, in particular, is generating a large quantity of pomace. The sour cherry pomace (SCP) is composed of the skin and remaining flesh, and its proportion varies between 15–28% of the original fruit mass, depending on factors such as the type of fruit, cultivation conditions and juice extraction method [2]. According to the literature, pomace is often used as feed, fuel and raw material for the isolation of bioactive compounds or discarded as waste [2]. However, due to the high content of valuable bioactive compounds (e.g., polyphenols) and functional components (e.g., dietary fibers (DFs) and pectin) [2,3], SCP could be utilized as an alternative and cheap raw material for the development of novel food products.

Fruit fillings (FFs) have been traditionally used for centuries as an ingredient for various bakery and confectionery products. In modern times, during which take-away snacks and fast-made meals are more often consumed, the production of bakery products filled with different fruit-based ripples is one of the most rapidly expanding food industries [4]. Even though FFs are often consumed in today’s day and age, they are still understudied food products [5]. Bakery FFs are generally made from some form of fruits, water, sugar, stabilizer and thickener [4]. Fruit ripples are considered a binary phase food system, whereas fruit particles are dispersed in a colloidal phase of carbohydrates (e.g., starch, pectin, xanthan, sugars, etc.), salts and acidity regulators, which are dissolved in water [6]. Even though this industry is expanding, consumers’ increasing demand for “healthier” food products and higher demand for food due to the rising human population have forced both the scientific community and the food industry to develop new food products and more sustainable food production with less discarded food waste. As a result of such trends, Carcelli et al. [7] proposed the usage of corn-based fiber syrup as a substitution for sugar in FFs. Young et al. [8] developed a novel polyuronan blend made from alginate and pectin whose synergistic effect enables stable texture and consistency of FFs during the cookie-baking process. Agudelo et al. [5] examined the thermal stability and syneresis of the tapioca starch–pectin model in one study, while viscoelastic properties and sensory characteristics were evaluated in a second study [9]. However, to the best of our knowledge, there are no studies attempting to tackle sustainable FFs production. One of the paths to integrate principles of a circular economy (e.g., reusing and recycling) with the food industry is to reuse residual fruit pomace after a juice extraction as a raw material for FFs, which was one of the main novelties and goals of the present study. If this is achieved, the present study could potentially inspire the scientific community and the food industry to further develop protocols involving residual fruit pomace as an ingredient for FFs.

The quality of bakery FFs depends on many properties and is regulated by many criteria such as food safety (microbiology, pesticide residuals, etc.), chemical composition (proximate analysis, mineral composition, bioactive compounds, etc.) [7], quality properties (syneresis and thermal properties, rheological properties, etc.) [4,7,8,10] and sensory characteristics [9] or consumer preference. To properly develop and test a new bakery FF which could be competitive in the food market, all aforementioned aspects needed to be tackled and achieved in one product. Therefore, another goal of the present study is to apply a holistic approach in the development of a new bakery sour cherry pomace filling (SCPF), evaluating the majority of important quality parameters for this type of food product and directly comparing SCPF with commercial sour cherry filling (CSCF). Since laboratory trials may be misleading, SCPF and CSCF were produced on a semi-industrial scale. The aim of this goal is to provide further insight into the impact of the production process closer to the industrial level on FFs quality, which to our best knowledge, was not reported in the literature.

## 2. Materials and Methods

### 2.1. Sample Preparation

#### 2.1.1. Filling Production

CSCF and SCPF were produced on a semi-industrial scale in the d.o.o. Nutri Sweet facilities (Novi Sad, Serbia). The former was produced with 30% (*w*/*w*) sour cherry (SC) berries, while the latter was made of 30% (*w*/*w*) SCP. Other ingredients included sugar (20–30%; *w*/*w*), water (30–40%; *w*/*w*), modified starch (10%; *w*/*w*), citric acid (0.1%; *w*/*w*) and potassium sorbate (<1000 mg/kg of sample), whereas exact concentrations of sugar and water are confidential and a trade secret of the d.o.o. Nutri Sweet. FF ingredients were mixed in a duplicator and thermally treated (80–90 °C) for 30 min to achieve complete gelatinization of samples. Freshly prepared samples were then transferred to plastic containers previously treated with aqueous ethanol (70% *w*/*w*) and left for cooling for 24 h at room temperature. Cooled samples were then submitted for further analysis.

#### 2.1.2. Baking Process

Dough for pastry was prepared in bakery pilot unit located at the Institute of Food Technology, Novi Sad, Serbia. Ingredients for the dough were white wheat flour (2 kg; wheat flour extraction rate ≤ 72%), yeast (75 g), salt (40 g), sucrose (80 g) and water (1.2 L) for each sample. All ingredients were measured and transferred to a dough mixer, homogenized for 7 min and left to rest for 5 min. The prepared dough was thinned into a 3 mm layer, cut into smaller pieces (7 cm × 10 cm, ~38–40 g), filled with CSCF or SCPF (~16–18 g), folded and left to ferment at room temperature for 45 min. Baking was performed in a convection oven (MDCOB18, Mac Pan, Thiene, Italy) at 240 °C for 15 min. Baked samples were left to rest at room temperature for 12 h, placed in plastic containers with no odor and distributed to conduct the consumer test.

### 2.2. Product Safety

#### 2.2.1. Microbiological Safety

Presence of *Salmonella* spp. [11], *Listeria monocytogenes* [12], *E. coli* [13], *Enterobacteriaceae* [14], yeasts and molds [15] in samples were determined by International Organization for Standardization (ISO) methods.

#### 2.2.2. Pesticides Content

Extraction of pesticides from SC bakery fillings was performed according to a slightly modified method reported by Kecojević et al. [16]. Firstly, samples were ground and homogenized in a coffee mill (TSM6A011W, Bosch, Gerlingen, Germany), and 10 g of samples were then measured into 50 mL plastic centrifuge tubes. Afterwards, 100 μL of internal standard (3-phenyl phosphate and nicarbazin; 10 mg/L) and 9.9 mL of cold acetonitrile were added to a tube. Samples were then vortexed for 5 min (Classic, Velp Scientifica, Usmate, Italy) and mixed with QuEChERS Mix (g magnesium sulphate, 1 g sodium chloride, 0.5 g disodium hydrogen citratesesquihydrate, 1 g sodium citrate dihydrate). A tube with the sample was again vortexed for 5 min and centrifuged (5804 R, Eppendorf, Hamburg, Germany) for 5 min at 5000 rpm. In total, 5 mL of extract was transferred in a 15 mL plastic tube, mixed with a clean-up mixture (900 mg MgSO_4_, 150 mg PSA, 150 mg C18), vortexed for 30 s and centrifuged for another 5 min at 5000 rpm. Additionally, 1 mL of obtained extract was mixed with 10 μL 5% formic acid aqueous solution. Prior to injection into LC-MS/MS and GC-MSMS systems, final extracts were filtered through polytetrafluoroethylene syringe filters (0.45 µm).

GC analysis was performed using (Trace 1300 GC, Thermo Fisher Scientific, Waltham, MA, USA) coupled with triple quadrupole mass spectrometer and electron ionization (EI) source (TSQ9000-NOVPI, Thermo Fisher Scientific, Waltham, MA, USA). Samples were inserted (1 µL) into GC-MS/MS system via programmable temperature vaporizer (PTV) injector (AI/AS1310 PTV, Thermo Fisher Scientific, Waltham, MA, USA) in a splitless mode (split flow 50 mL/min) with temperature set to 90 °C. Mobile phase was helium (99.999%) with a flow rate set to 1.2 mL/min. Pesticides were separated on a TG-5SiMS (30 m × 0.25 mm × 0.25 μm, Thermo Fisher Scientific, Waltham, MA, USA) analytical column. The GC oven temperature gradient was programmed as follows: initial temperature was 70 °C; hold for 1.5 min at 70 °C; temperature increase to 90 °C with rate 25 °C/min; hold for 1.5 min at 90 °C; temperature increase to 180 °C with rate 25 °C/min; temperature increase to 280 °C with rate 5 °C/min; temperature increase to 300 °C with rate 10 °C/min; and hold for 10 min at 300 °C. Electron ionization voltage was set to 70 eV while filament current was set to 50 µA. The optimum multiplier optimum voltage was examined by the autotune procedure with perfluorotributylamine solution. Argon, with 99.9998% purity, was the collision gas. Temperatures of the transfer line and EI source were set to 280 and 300 °C, respectively.

LC analysis was performed using (Vanquish Core, Thermo Fisher Scientific, Waltham, MA, USA) coupled with triple quadrupole mass spectrometer and HESI source (TSQ Quantis, Thermo Fisher Scientific, Waltham, MA, USA). Pesticides were separated on AccucoreTMaQ (100 × 2.1 mm × 2.6 µm, Thermo Fisher Scientific, Waltham, MA, USA) analytical column. Mobile phase A was water:methanol:formic acid (v:v:v 97.9:2:0.1%) with 5 mM ammonium formate, while mobile phase B was water:methanol:formic acid (v:v:v 2:97.9:0.1%) with 5 mM ammonium formate. Mobile phase program was as follows: 0–0.5 min, 2%B; 0.5–2 min 40%B, 2–20 min 95%; 20–23 min 95%B; 23–23.1 2%B; and 23.1–27 min 2%B. Sample volume injection was 5 µL, flow rate was 0.3 mL/min, and column compartment temperature was set to 35 °C. The detector operated in a positive and negative mode with positive and negative ion spray voltage set to 3700 and 2500 V, respectively. Sheath gas, aux gas and sweep gas were 30, 6 and 0 Arb, respectively. Ion transfer tube temperature was 300 °C, while vaporizer temperature was 280 °C. Argon, with 99.9998% purity, was the collision gas, with collision gas pressure set to 1.5 mTorr.

Scan type for LC and GC system was Selected Reaction Monitoring (SRM). Data acquisition and assessment were performed using TraceFinder program (version 5.1, Thermo Fisher Scientific, Waltham, MA, USA). Calibration curves were constructed with analytical standards of all pesticides listed in Table S1. The results were expressed as milligrams of pesticide per 1 kg of sample fresh weight (mg/kg fw).

### 2.3. Chemical Composition and Bioactivity

#### 2.3.1. Proximate Analysis

Moisture (984.25), ash (925.51), protein (950.36), fat (935.38) and dietary fiber (985.29) content of FFs were determined by standard methods defined by the Association Official of Analytical Chemists [17]. FFs sugar concentration was determined by Luff–Schoorl method [18], while carbohydrate content was calculated by subtraction of moisture, ash, protein and fat content from 100.

#### 2.3.2. Mineral Composition

Zn, Cu, Fe, Mg, K, Na, Ca and Mn content in FFs was determined by atomic absorption spectroscope (iCE 3300 Fl, Thermo Fisher Scientific, Waltham, MA, USA) according to Perović [19] method.

#### 2.3.3. Total Polyphenol Content and Individual Anthocyanin Content

Extraction of polyphenols was performed according to modified method reported by Wu et al. [20]. Fresh SC bakery fillings were ground and homogenized. Then, 1 g of sample was transferred in conic plastic tube and with 10 mL of MeOH:H_2_0:HCl (v:v:v; 85:14.5:0.5%), vortexed for 30 s and sonicated in an ultrasound bath (VTUSC6, Velleman, Gavere, Belgium) for 5 min, again vortexed for 30 s, sonicated for 5 min and left for 10 min at room temperature. Obtained samples were centrifuged for 5 min at 5000 rpm, supernatant was separated while solid part was again treated with 5 mL of MeOH:H_2_O:HCl and the whole procedure was repeated. Afterwards, supernatants were mixed, and 8 mL of obtained extract was dried in N_2_ atmosphere. Prior to analysis, dry extracts were re-dissolved in 2 mL of methanol.

Total polyphenol content (TPC) of prepared extracts was analyzed by Folin–Ciocalteu assay [21]. Absorbance was scanned at 750 nm using spectrophotometer (Shimadzu UV-1800, Kyoto, Japan), and all experiments were performed in triplicate. The results were obtained with previously constructed calibration curve (R^2^ = 0.991), and mean values of TPC of prepared samples were expressed as grams of gallic acid equivalents (GAE) per 100 g of sample fresh weight (g GAE/100 g fw).

Anthocyanin content was determined according to slightly modified HPLC/DAD reported by Wu et al. [20]. Briefly, previously prepared extracts were filtered through regenerated cellulose syringe filter (0.45 µm) and injected into HPLC/DAD system (1200 Series, Agilent, Santa Clara, CA, USA) with Zorbax Eclipse XDB-C18 (4.6 mm × 50 mm × 1.8 µm) analytical column. Anthocyanins were separated using 1% formic acid aqueous solution as mobile phase A and methanol as mobile phase B. Other parameters, such as flow rate, wavelength and gradient program (1), were set as reported by Wu et al. [20]. Calibration was performed with cyanide-3-O-glucoside analytical standard, and results were expressed as milligrams of cyanide-3-O-glucoside equivalent (CGE) per 100 g of sample fresh weight (mg CGE/100 g fw).

#### 2.3.4. Antioxidant Activity

Antioxidant activity of obtained extracts (Section 2.3.3) was determined using a DPPH assay [21]. The methanol solution of the DPPH free radical was freshly prepared (absorbance of 0.70 ± 0.02). The volume of 100 µL of properly diluted extracts was mixed with 2.9 mL of DPPH free radical solution. The samples were then incubated at room temperature in the dark for 1 h. Antioxidant activity measurements were performed in triplicates at 517 nm (Shimadzu UV-1800, Kyoto, Japan). The results were reported as µmol of Trolox equivalents (TE) per g of sample fresh weight (µmol TE/g fw).

### 2.4. Quality Properties

#### 2.4.1. Textural Properties

Hardness/firmness, work of shear, stickiness and work of adhesion of FFs were determined on a texture analyzer TA.XTplus (Stable Micro Systems, Godalming, UK) according to Belović et al. [22] method.

#### 2.4.2. Rheological Properties

Storage modulus (G′) and loss modulus are determined on rheometer (Haake Rheo Stress 600HP, ThermoFisher Scientific, Dreieich, Germany) coupled with parallel plate geometry PP35 using temperature sweep test (25 °C–90 °C) at a fixed stress (1 Pa) and frequency (1 Hz) [22].

#### 2.4.3. Syneresis

Syneresis of fillings before baking process was tested by centrifuging (Eppendorf, 5804R, Hamburg, Germany) 10× *g* of samples at 14,880 g (11,000 rpm) during 30 min at 20 °C and visual examination of sample’s surface for eventual water migration.

#### 2.4.4. Thermal Stability

CSCF and SCPF were applied on baking paper in 1 cm layer with a diameter of 5 cm using metal mold. Prepared samples were then baked in a convection oven at 100 °C for 10 min mimicking approximate conditions during the baking process. Differences in diameter between fresh and baked samples were measured with Vernier calliper.

### 2.5. Questionnaire and Sensory Testing

Consumers were recruited among students, teachers and workers from the University of Novi Sad in Serbia. Initial contact with potential participants was established by phone or email to determine eligibility, and if eligible, an appropriate evaluation time was agreed upon. A total of 56 consumers participated, of whom 35 were female and 21 male. The age of the respondents ranged from 18 to 65 years. The participants were provided with information about the study, and those who agreed to take part in the testing process signed an informed consent form. The entire sensory test was performed via online questionnaire, which is presented in the Supplementary Materials.

**Figure 1:**
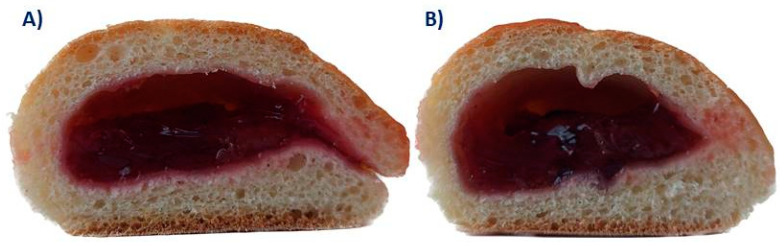
Cross section of baked pastry with (**A**) commercial sour cherry filling and (**B**) sour cherry pomace filling.

Two samples (Figure 1) considered in the study were presented to the consumers in closed, odorless plastic containers labelled with three-digit random numbers at room temperature. To identify hedonic liking, respondents were asked to taste two individual samples and indicate their liking for appearance, odor, taste, aroma and texture using a 5-point hedonic scale (from 1 = unacceptable to 5 = extremely acceptable). To identify the consumers’ liking of the analyzed pair of samples, they were asked to decide which one they preferred in relation to the quality of the filling. To answer the question about product preferences, they had to select only one product out of the two they had tested. In addition, they were asked to indicate the extent to which they would be likely to purchase or refuse to buy the offered products.

### 2.6. Statistical Analysis

Analytical measurements were mainly performed in at least three repetitions (unless otherwise stated), and the results were presented as mean value ± standard deviation. The one-way analysis of variance (ANOVA) was used to determine whether there were any statistically significant differences between the means of independent samples (*p* < 0.05). Statistical analysis was performed with STATISTICA 13.0 (StatSoft, Palo Alto, CA, USA).

## 3. Results

### 3.1. Semi-Industrial Processing of Sour Cherry Bakery Fillings

**Figure 2:**
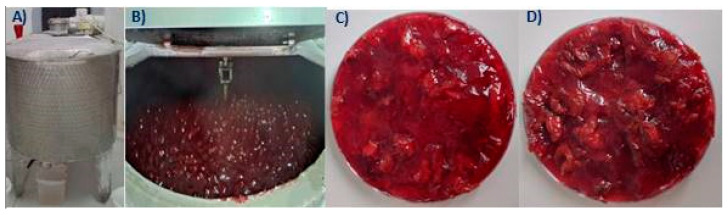
Semi-industrial production facilities for sour cherry fillings (**A**,**B**); commercial sour cherry filling (**C**); sour cherry pomace filling (**D**).

Prior to the commercialization of any food product, it is required to scale up the process on a semi-industrial level because laboratory conditions might differ from semi-industrial and could need additional adjustments in order to optimize food production. Therefore, in order to properly test the possibility of using SCP as a raw material for bakery fillings, commercial sour cherry filling (CSCF) and SCPF were produced in the same semi-industrial conditions (Section 2.1, Figure 2).

Furthermore, prior to market placement, the novel food product SCPF needs to satisfy a wide range of food safety, nutritional, quality and sensory parameters.

### 3.2. Product Safety

There is an increasing trend within the food industry to reduce food waste and recycle or reuse its by-products in order to align with European Circular Economy Action Plan One. Various food by-products could be valuable since they contain nutrients and bioactive compounds; however, prior to their further use, issues of food safety should be thoroughly evaluated [23]. These food safety issues are mainly related to the presence of microorganisms that cause food spoilage and pathogens and the presence of food contaminants (residues of pesticides, mycotoxins, etc.) [23]. Food safety parameters determined for SC, SCP, CSCF and SCPF are presented in Table 1. Due to the relatively high content of moisture in SCP, it could be susceptible to microbial spoilage. *Salmonella* spp. and *Listeria monocytogenes* were not detected in any of the tested samples, while *E. coli* concentration was below the limit of quantification (<10 cfu/g) in all samples. On the other hand, *Enterobacteriaceae*, yeasts and molds were detected in 10- and 20-fold higher concentrations in the filling’s raw material compared to the final products (Table 1). However, the concentration of these microorganisms in the final products was below <10 cfu/g, which indicates that the thermal process during SC fillings production was performed appropriately, and fillings could be considered microbiologically safe.

Pesticides are commonly used to protect plants from other plants, microorganisms, insects and animals and thus increase crop yield. However, as it is well-known, pesticides are harmful to human health, and the concentration of pesticides should be determined in all food products. In total, 176 and 326 pesticide residues were evaluated by GC-MS/MS and LC-MS/MS, respectively (Table S1). The concentration of all pesticides besides acetamiprid was below the limit of quantification (<0.005 mg/kg). Acetamiprid is one of the most important pesticides for SC cultivation, which is applied on cherry trees before bloom [24]; thus, it can sometimes be found in cherry berries [25]. The concentration in SCPF (0.035 mg/kg) was significantly higher compared to CSCF (0.006 mg/kg). This indicates that the majority of pesticide was accumulated on the fruit skin, which has a larger mass fraction in pomace compared to the entire berry. However, it is important to emphasize that in both samples, acetamiprid was detected in at least 40-fold lower concentration than the allowed maximum residual level for cherries (1.5 mg/kg) defined by the European Commission (EC); thus, CSCF and SCPF were safe for consumption in this regard.

### 3.3. Chemical Composition and Bioactivity

The content of nutrients and bioactive compounds such as proteins, DFs, minerals and polyphenols represents an important quality parameter in many food products, including bakery FFs. Differences in the proximate composition of final products (CSCF and SCPF) were minimal (<1 g/100 g fw) apart from moisture content (MC) and dietary fiber content (DFC) (Table 2). Higher moisture content in CSCF could be explained by differences in fruit raw material (Section 2.1), as pitted cherries were used for CSCF while pomace was a fruit raw material for SCPF. This is aligned with the literature since the MC of fresh SC is approximately 85% [26], while approximately 75% of MC was determined in SCP [27]. Consummation of DFs is associated with several health benefits, including blood pressure and sugar level regulation, a decrease in total and LDL cholesterol, etc. [28]. Therefore, DFs are a crucial element for a healthy diet, and the current direction of the food industry is oriented towards enhancing the nutritional content of final products by adding fibers. Since SC DFs are mainly present in the skin, it was rather expected that SCPF (3.79 ± 0.02 g/100 g fw) would have significantly higher DFC than CSCF (0.79 ± 0.01 g/100 g fw). Thus, the usage of pomace fillings with higher DFC could not only be beneficial from an economic point of view but could also greatly contribute to the overall functionality of bakery products. It is noteworthy that due to the increase of DFC in SCPF, this pomace filling could be classified as a “source of fiber”, since, according to EC Regulation (No. 1924, 2006), the minimal content of fiber in food products to achieve such classification is 3 g/100 g.

Minerals belong to a group of essential nutrients that are consumed in relatively small quantities compared to macronutrients but are equally as important for human health. These inorganic nutrients have a wide range of activities in the human body which were thoroughly reviewed by Soetan et al. [29]. The most abundant mineral in CSCF and SCPF was Na (863.65–1107.40 mg/kg fw), while Cu (0.31–0.54) and Mn (0.41–0.42 mg/kg fw) were detected in the lowest concentrations. It is important to stress that the majority of determined minerals which can be beneficial for human health were detected in a higher concentration in filling with pomace compared to filling with the pitted fruits (Table 2). In particular, Fe is important for oxygen transportation in blood via hemoglobin [29], and Fe content in SCPF and CSCF was 3.83 and 2.87 mg/kg fw, respectively. Zn is a cofactor and constituent for many enzymes in the human body [29], and its concentration was 1.44 and 0.99 mg/kg in SCPF and CSCF, respectively. Cu, which is essential to bone development and helps in Fe incorporation in hemoglobin, was detected at an approximately 1.7-fold higher concentration in SCPF when compared to the control sample. The obtained results indicated that the majority of minerals were stored in the outer layers of the SC berry, specifically in the skin. Thus, the incorporation of SCP in bakery filling could additionally improve and fortify the functionality of the proposed novel food product.

Polyphenols are secondary metabolites that are synthesized by all higher plants and serve as plant protection against unfavorable growing conditions and various pests. These compounds are well-known for their antioxidant, antimicrobial, anticancer, anti-inflammatory and many other bioactivities. Diets rich in polyphenols were associated with protection against various chronic diseases (e.g., cardiovascular diseases, tumors, neurodegenerative diseases, etc.) [30], which are ubiquitous in the modern world. Thus, food products with high polyphenol content (e.g., SC) are in high demand in the global market. However, there are also studies with heterogeneous health responses to polyphenols-rich diets, which pointed out controversy in conclusions between clinic studies [31]. Polyphenols content in SC varies between cultivars, berry parts and growing conditions [32,33], and different phenolic compounds were detected in SC berries [34]. Anthocyanins are a subgroup of polyphenols that are abundant in SC and responsible for the fruit’s red color. One of the most abundant anthocyanins in SC is cyanidin-3-O-glucosyl-rutinoside (Cy-glc-rut) [33,34,35], and its concentration in CSCF and SCPF was 7.89 and 5.29 mg CGE/100 g fw, respectively (Table 2). The content of cyanidin-3-O-rutinoside (Cy-rut) was also lower in SCPF (2.29 mg CGE/100 g fw) in comparison to CSCF (3.41 mg CGE/100 g fw) while cyanidin-3-O-sophoroside was detected only in control filling (0.77 mg CGE/100 g fw). These findings were initially rather surprising since the majority of polyphenols, including anthocyanins, are accumulated in the skin of fresh SC berries [32], which has a higher mass fraction in SCPF than CSCF. However, a large number of anthocyanins might have been separated as a juice fraction from SCP during industrial processing. This is supported by Repajić et al.’s study [35], which reported that mass fractions of total monomeric anthocyanins content were approximately 2-fold higher in pressed and filtered juice in comparison to fresh fruits of Oblačinska and Marasca SC cultivars. Since FFs were made with 30% of fruit-based components and since only cherries contributed to anthocyanin content, it could be stated that Cy-glc-rut (approximately 12.3–18.4 mg CGE/100 g fw) and Cy-rut (approximately 5.3–8 mg CGE/100 g fw) content was in CSCF and SCPF. The concentration of Cy-glc-rut reported for five Hungarian SC cultivars was approximately 5–15 mg/100 g fw, which is somewhat aligned with the present study, while Cy-glc-rut was several folds lower in comparison to the presented study (Table 2) [36]. On the other hand, Cy-glc-rut and Cy-glc-rut concentration in the present study was significantly lower than in cv. Marasca (61 and 160 mg/100 g fw, respectively) and cv. Oblačinska (83 and 100 mg/100 g fw, respectively) [35]. Total phenolic content (TPC) was slightly higher in SCPF (152.40 mg GAE/100 g fw) in comparison to CSCF (140.73 mg GAE/100 g fw) with a lack of statistical differences. Similar to the concentration of individual anthocyanins, TPC reported for Marasca (1350 mg/100 g fw) and Oblačinska (1140 mg/100 g fw) varieties were several folds higher in comparison to TPC in the present study [35], which could be attributed to aforementioned reasons. Due to the presence of polyphenols, CSCF and SCPF had certain antioxidant activity measured by DPPH assay; however, there was a lack of statistical differences between the two tested samples (Table 2). The presence of antioxidants might have a role in shelf-life extension and preservation of sensory characteristics of FFs which are prone to deterioration by oxidation processes.

### 3.4. Quality Properties

FFs’ rheological properties are some of the key focuses in the production unit designing, production optimization and final product quality assurance as rheological properties provide information about FFs’ molecular structure, textural characteristics and sensory properties (mouthfeel and body). These properties are mainly driven by fruit type (e.g., SC, apple, forest fruits, etc.) and its content, starch and hydrocolloids (e.g., pectin, alginate, xanthan, gum arabic, etc.) content, sweeteners (e.g., sucrose, glucose, fiber syrup, etc.), acidifiers (e.g., citric acid, malic acid, etc.) content and production parameters [6].

Hardness/firmness, work of shear, stickiness and work of adhesion are commonly determined textural parameters for FFs or jams [22,37,38,39]. It is important to stress that determination of rheological properties was more difficult in comparison to other quality parameters (proximate analysis, pesticides, polyphenols analysis, etc.). This is due to the presence of skin particles (Figure 1), which were intact before but were ground and homogenized later. Grinding samples prior to rheological analysis is not appropriate since determined properties would not present a real food system. As a result, standard deviations were relatively large for rheological properties in comparison to other analyses (Table 2, Table 3 and Table 4). The statistical difference between CSCF and SCPF were determined by ANOVA for hardness/firmness and stickiness (Table 3). Hardness or firmness is defined as the maximum force on a filling that displays resistance to mechanical deformation [39]. Since SCPF has higher dry matter content in comparison to CSCF (Table 1), it was rather expected that SCPF would be a firmer sample. However, higher dry matter does not necessarily result in a higher firmness [22]. Higher amounts of sugars [22,37] and higher mass fraction of skins [39] in jams/fillings could also lead to firmer products, a finding which is aligned with the present study. DFC is another relevant factor for fillings hardness, as a higher quantity of DFs leads to a firmer product. This is aligned with a recent study where the addition of gold flax and chia seeds in cranberry jams significantly increased DFC and, consequently, the hardness of jams [40]. Stickiness is a textural property defined by the maximum force required to separate the probe from the sample and represents the extent of sample adhesiveness on the teeth during/after biting [37] and, together with work of adhesion, could be related to spreadability [22]. FFs/jams containing a higher quantity of pectin tend to exhibit greater adhesiveness and spreadability due to the increased incorporation of fruit solids into the pectin network [22]. Furthermore, since SCP contains up to 14.64% of pectin [41] while pectin quantity in fresh SC berries is in the range of 0–0.72% [42], it should be expected that SCPF is less sticky and it is more spreadable in comparison to CSCF (Table 3). However, this was not the case with SC fillings, which indicates that other components, such as sugars and fibers, also played an important role.

According to Basu and Shivhare [37], the work of shear is an accumulative amount of force necessary for the entire shearing process. Even though there was a lack of statistical differences between samples, SCPF exhibited higher work of shear in comparison to CSCF (Table 3). The explanation for such an outcome is similar to other textural properties. Apart from stickiness and work of adhesion, work of shear could also be a measure of spreadability [37]. Bakery FFs should generally be less spreadable products in comparison to jams. FFs are often applied as a thick layer only in the central parts of a pastry before the baking process, while the purpose of jam is to be easily spread on already-baked bread, cookies, pancakes, cakes, etc. Indeed, the work of shear of SC fillings (292.27 and 328.53 g s) was higher in comparison to mango jam with an approximately equal sugar content of 50 g/100 g (229.18 g s) [37] and tomato jam (0.043 g s) with a sugar content of 43.2 g/100 g [22]. Work of adhesion represents the total force required to separate the probe from a sample [37], and differences between the two samples were minimal (Table 3).

FFs are food systems that possess the elastic and viscous properties of fluids. Viscoelastic properties of FF can be represented by storage modulus (G′) and loss modulus (G″). In practical terms, G′ is a measure of reversible (elastic) changes in food structure, while G″ is a measure of irreversible (viscous) changes in fillings structure [22]. G’ values are approximately 4-fold higher in comparison to G″ values for both samples at the same experimental conditions (Table 4). This indicates that SC fillings have a weak gel structure and that fillings behave more similarly to a solid system rather than a viscous system [43]. Storage and loss modulus is dependent on temperature, whereas the increase in temperature leads to a decrease in both modulus values [43,44], and this is aligned with the presented study. Furthermore, G′ and G″ values obtained at the same temperature are approximately 1.3–1.5 higher for SCPF in comparison to CSCF, suggesting that chemical composition played a role in viscoelastic properties as well. Higher pectin content and more developed gel structure [45] within SCPF could be responsible for such an outcome. Additionally, DFC in SCPF (Table 1) could result in higher G′ and G″ values [45].

The term syneresis can be used to describe the separation of liquid from a gel or a colloidal system over time, leading to the formation of a liquid layer on top of the gel or the colloidal matrix. Syneresis could be prevented by equilibrating water concentration between filling phases. This equilibrium could be achieved by the selection of filling formulations with high soluble solid content [8] and/or by the addition of different hydrocolloids (e.g., pectin and gellan gum) in fillings [4]. Pectin is one of the most commonly used gelling agents in FFs; however, the pectin’s colloidal structure in FFs is not resistant to mechanical stress, which could damage the pectin network and lead to leakage of fruit ripples from pastry products. Therefore, pectin, which is naturally present in SC and SCP [2], should be combined with a thickening agent to avoid syneresis [6]. Indeed, obtained results suggested that 10% modified starch (Section 2.1) and pectin from SC and SCP were sufficient to prevent any water migration on the surface of CSCF and SCPF (Table 3). This is also important from a food safety point of view since a high quantity of free water on the surface of the filling could result in undesirable microbiological spoilage.

**Figure 3:**
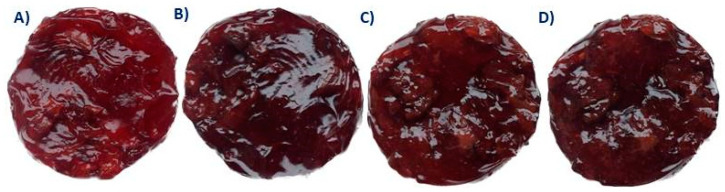
Commercial sour cherry filling before (**A**,**B**) and after baking at 100 °C; sour cherry pomace filling before (**C**,**D**) and after baking at 100 °C.

The thermal stability of CSCF and SCPF was evaluated by baking samples in an oven at 100 °C. Testing thermal stability is also of paramount importance for FFs since they are used as an ingredient for pastry products and could leak from dough at high temperatures during the baking process [10]. Both CSCF and SCPF exhibited good thermal stability since differences between the initial diameter of the applied filling (5 cm) and diameter after baking at 100 °C were minimal (5.12 and 4.95 cm, respectively). It is noteworthy that water migration to the product surface was not detected during the baking process as well, suggesting that, indeed, the gel structures of fillings were appropriately formed (Figure 3). Thus, CSCF and SCPF are suitable to be applied in the bakery industry.

### 3.5. Questionnaire and Sensory Testing

Evaluation of consumers’ acceptance and liking for a certain food is of paramount importance for the development of novel food and has been broadly utilized to measure consumers’ hedonic response to a product [46]. In certain cases, novel food can be enriched with essential nutrients (e.g., polyphenols, vitamins, minerals, DFs, etc.) and still be non-competitive on the food market due to a lack of consumer preference towards that food product. Therefore, some kind of consumer test should be performed for each novel food prior to any commercialization. A group of 56 participants was asked to perform a consumer test of pastries with CSCF and SCPF.

Five basic sensory properties (appearance, odor, taste, aroma and texture) were evaluated in the first test. According to the results, all five sensory properties for both pastries with SC fillings were rated on average with >4 of a maximum of 5, with the exception of SCPF texture (Table 5). Even though there was a lack of statistical difference between pastries with CSCF and SCPF for all five tested properties, it is noteworthy to point out that pastries with SCPF had higher average ratings in odor and aroma. Suggesting that a higher fraction of SC skins might have enhanced cherry aroma and odor in pastry with SCPF. Furthermore, the average rating was equal for both samples.

In the second test, participants were asked whether they preferred a sample with CSCF, pastry with SCPF, both or neither, and in total, 16, 19, 21 and 0 participants, respectively, selected the aforementioned options (Figure S1). This further supports the conclusions that both pastries were generally acceptable for consummation from a sensory point of view and that there was a lack of clear differences in consumer preference between tested samples. In the third part of the sensory evaluation, participants were asked to rate if pastries with fillings were attractive or repulsive to them. The majority of participants (46 for CSCF and 48 for SCPF) answered that pastries were attractive to some extent, while the minority was indecisive about whether they enjoyed the tested samples or not (7 for CSCF and 10 for SCPF) (Figure S2). As the last part of the sensory evaluation, participants were asked about preferences between two samples. Considering the results obtained in the first part of the study, it is rather expected that there was a lack of differences in consumer preference between the two samples, as 28 participants preferred each of the tested pastries (Figure S1). Due to all of the aforementioned points, it can be concluded that SCP could be utilized as a raw material for FF, which will be highly competitive with commercial FFs due to its low price.

## 4. Conclusions

A comprehensive study was performed to thoroughly test and compare novel SCPF with CSCF. This approach included the evaluation of food safety, chemical composition, bioactivity, textural properties, rheological properties and sensory characteristics. The results suggested that both samples were safe for human consumption since all tested microorganisms and pesticides were determined to have quantities lower than maximally allowed in food products. DFC in SCPF was significantly higher in comparison to CSCF, and it could be considered a “source of fibers”. SCPF was also superior in terms of mineral composition since filling with pomace had a higher skin fraction which is richer in minerals. The concentration of individual anthocyanins was lower in SCPF, suggesting that a significant amount of these polyphenols were removed from SC skin during juice extraction. Both samples were thermally stable, and there was a lack of syneresis, indicating that the colloidal network of fillings was stable. Textural and rheological parameters suggested that CSCF is generally more spreadable and less stiff due to the lower content of fibers, pectin, sugars, etc. However, SCPF was still appropriate for the intended use. According to the consumer test, the pastry with SCPF was practically equally preferred as the pastry with CSCF. Therefore, SCP could be used as a raw material for bakery FFs production on an industrial scale which could result in a decrease in food waste discarded by the SC juice industry. Additionally, the usage of SCP instead of the entire SC berries by the fillings industry leads to a significant reduction in production cost. Since the new SCPF is cheaper in comparison to CSCF, has acceptable quality and sensory properties when produced on a semi-industrial scale and is safe for human consumption, it could be competitive with other FFs in the food market. Future research should be focused on (I) scaling the process from the semi-industrial to the industrial level and (II) an economic study on the industrial unit necessary to evaluate the true cost of SCPF production. Additionally, other fruit pomaces could be tested as raw materials for fruit fillings. Some of the limitations of SCPF production could be competitiveness for raw materials between the FF industry and other industries that could use SCP (e.g., the animal feed industry).

## Figures and Tables

**Table 1:** Food safety parameters determined for sour cherry fillings.

Microorganism	Sour Cherry	Sour Cherry Pomace	CSCF	SCPF
*Salmonella* spp./25 g	n.d.	n.d.	n.d.	n.d.
*Listeria monocytogenes*/25 g	n.d.	n.d.	n.d.	n.d.
*E. coli* (cfu/g)	<10	<10	<10	<10
Enterobacteriaceae (cfu/g)	100	110	<10	<10
Yeasts and molds (cfu/g)	200	220	<10	<10
**Pesticide** (mg/kg fw)				
Acetamiprid *	n.a.	n.a.	0.006 ± 0.00 ^a^	0.035 ± 0.001 ^b^
All other pesticides	n.a.	n.a.	<0.005	<0.005

The results are presented as the value of one analysis, *n* = 1, * or as mean ± SD, *n* = 3. Different letters (^a^ and ^b^) within the same row indicate significant statistical differences according to ANOVA test (*p* < 0.05). CSCF—commercial sour cherry filling; SCPF—sour cherry pomace filling; n.d.—not detected; n.a.—not analyzed; fw—fresh weight.

**Table 2:** Proximate mineral composition and polyphenols composition and antioxidant activity of sour cherry bakery fillings.

Proximate Composition (g/100 g fw)	CSCF	SCPF
Moisture	52.62 ± 0.02 ^b^	48.36 ± 0.03 ^a^
Ash	0.31 ± 0.02 ^a^	0.30 ± 0.02 ^a^
Carbohydrates of which	45.76 ± 0.70 ^a^	46.87 ± 0.65 ^a^
Sugars	28.81 ± 0.50 ^a^	29.72 ± 0.05 ^b^
Dietary fibers	0.79 ± 0.01 ^a^	3.79 ± 0.02 ^b^
Proteins	0.49 ± 0.03 ^a^	0.59 ± 0.03 ^b^
Fats	0.03 ± 0.00 ^a^	0.04 ± 0.00 ^a^
Salt	0.04 ± 0.01 ^a^	0.05 ± 0.01 ^a^
Total soluble solids (°Brix)	43.38 ± 0.03 ^a^	44.86 ± 0.07 ^b^
**Mineral composition** (mg/kg fw)		
Zn	0.99 ± 0.05 ^a^	1.44 ± 0.02 ^b^
Cu	0.31 ± 0.01 ^a^	0.54 ± 0.02 ^b^
Fe	2.87 ± 0.04 ^a^	3.83 ± 0.09 ^b^
Mg	62.64 ± 1.70 ^a^	85.77 ± 2.01 ^b^
K	1107.40 ± 7.84 ^a^	863.65 ± 5.06 ^b^
Na	164.63 ± 2.03 ^a^	206.72 ± 3.94 ^b^
Ca	97.37 ± 1.08 ^a^	173.03 ± 2.80 ^b^
Mn	0.41 ± 0.02 ^a^	0.42 ± 0.01 ^a^
**Polyphenols composition and antioxidant activity**		
Total polyphenol content (mg GAE/100 g fw)	140.73 ± 3.59 ^a^	152.40 ± 6.39 ^a^
DPPH (µmol TE/g fw)	20.65 ± 1.00 ^a^	21.33 ± 0.24 ^a^
Cyanidin-3-O-sophoroside (mg CGE/100 g fw)	0.77 ± 0.03	n.d.
Cyanidin-3-O-glucosyl-rutinoside (mg CGE/100 g fw)	7.89 ± 0.19 ^b^	5.29 ± 0.06 ^a^
Cyanidin-3-O-rutinoside (mg CGE/100 g fw)	3.41 ± 0.02 ^b^	2.29 ± 0.08 ^a^
∑ Anthocyanins (mg CGE/100 g fw)	12.09 ± 0.17 ^b^	7.58 ± 0.13 ^a^

The results are presented as mean ± SD, *n* = 3. Different letters (^a^ and ^b^) within the same row indicate significant statistical differences according to ANOVA test (*p* < 0.05). CSCF—commercial sour cherry filling; SCPF—sour cherry pomace filling; n.d.—not detected; fw—fresh weight; GAE—gallic acid equivalent; TE –trolox equivalent; CGE—cyanidin-3-O-glucoside equivalent.

**Table 3:** Syneresis, thermal stability and textural properties of sour cherry bakery fillings.

Parameter	CSCF	SCPF
Hardness/firmness (g force) *	319.90 ± 22.76 ^a^	354.88 ± 10.25 ^b^
Work of shear (g s) *	292.27 ± 46.06 ^a^	328.53 ± 29.88 ^a^
Stickiness (g force) *	−209.42 ± 17.96 ^b^	−251.54 ± 7.25 ^a^
Work of adhesion (g s) *	−144.21 ± 5.85 ^a^	−142.64 ± 5.75 ^a^
Syneresis	n.d.	n.d.
Diameter of filling after baking at 100 °C (cm)	5.12 ± 0.19 ^a^	4.95 ± 0.06 ^a^

The results are presented as mean ± SD, * *n* = 4, *n* = 3. Different letters (^a^ and ^b^) within the same row indicate significant statistical differences according to ANOVA test (*p* < 0.05). CSCF—commercial sour cherry filling; SCPF—sour cherry pomace filling; n.d.—not detected.

**Table 4:** Viscoelastic properties of sour cherry fillings obtained at constant frequency (1 Hz), stress (1 Pa) and different temperatures (25–90 °C).

Parameter	Temperature (°C)	CSCF	SCPF
G′ (Pa)	25	919.4 ± 19.7 ^a^	1170 ± 45.6 ^b^
	60	867.6 ± 13.5 ^a^	1159 ± 30.3 ^b^
	90	797.9 ± 2.5 ^a^	1029 ± 15.5 ^b^
	60	903.9 ± 20.4 ^a^	1181 ± 30.5 ^b^
	25	884.8 ± 25.6 ^a^	1339 ± 31.7 ^b^
G″ (Pa)	25	155.4 ± 10.2 ^a^	218.4 ± 23.5 ^b^
	60	174.4 ± 12.9 ^a^	227.2 ± 6.5 ^b^
	90	149.7 ± 0.6 ^a^	224.9 ± 10.2 ^b^
	60	183.4 ± 7.6 ^a^	205.9 ± 19.6 ^b^
	25	174.5 ± 18.5 ^a^	236.9 ± 19.5 ^b^

The results are presented as mean ± SD, *n* = 3. Different letters (^a^ and ^b^) within the same row indicate significant statistical differences according to ANOVA test (*p* < 0.05). G′—storage modulus; G″—loss modulus; CSCF—commercial sour cherry filling; SCPF—sour cherry pomace filling.

**Table 5:** Consumer test of pastry with commercial sour cherry filling (CSCF) and sour cherry pomace filling (SCPF).

	CSCF	SCPF
Appearance	4.39 ± 0.74 ^a^	4.12 ± 0.78 ^a^
Odor	4.02 ± 0.84 ^a^	4.08 ± 0.74 ^a^
Taste	4.12 ± 0.78 ^a^	4.25 ± 0.63 ^a^
Aroma	4.06 ± 0.84 ^a^	4.18 ± 0.63 ^a^
Texture	4.00 ± 0.90 ^a^	3.96 ± 0.71 ^a^
Average score	4.12 ± 0.70 ^a^	4.12 ± 0.83 ^a^

The results are presented as mean ± SD, *n* = 56. Same letter (^a^) within the same column indicate lack of significant statistical differences according to ANOVA test (*p* < 0.05).

## Data Availability

Data is contained within the article and supplementary material.

## Supplementary Materials

The following supporting information can be downloaded at https://www.mdpi.com/article/10.3390/antiox12061234/s1, Figure S1: Preference of sour cherry fillings. Test 2 in blue, test 4 in red; Figure S2: Attractiveness of pastries with (a) commercial sour cherry filling (b) sour cherry pomace filling. Table S1: The list of pesticides determined with LC-MS/MS and GC-MS/MS at LOQ = 0.005 mg/kg; Questionnaire—assessment of pastries filled with cherry filling.
