# Supplementary material for: Sour Cherry Pomace Valorization as a Bakery Fruit Filling: Chemical Composition, Bioactivity, Quality and Sensory Properties
Source: Antioxidants (Basel). 2023 Jun 7;12(6):1234. doi: 10.3390/antiox12061234 (PMC10294872; doi:10.3390/antiox12061234)
Supplement: Supplementary file 1 [file antioxidants-12-01234-s001.zip › antioxidants-2432681-supplementary.pdf]

Supplementary material

Table S1. The list of pesticides determined with LC-MS/MS and GC-MS/MS at LOQ=0.005 mg/kg

	GC-MS/MS		LC-MS/MS	
	Pesticide	R^2	Pesticide	R^2
1	2,4,5-T-Methylester	0.9948	Acephate	0.9958
2	2,4-D-Methylester	0.9971	Acetamiprid	0.9972
3	Acetochlor	0.9982	Alachlor	0.9946
4	Aclonifen	0.9992	Aldicarb	0.9972
5	Acrinathrin	0.9989	Aldicarb sulfone	0.9965
6	Azoxystrobin	0.9968	Aldicarb sulfoxide	0.9973
7	Aldrin	0.9956	Ametoctradin	0.9968
8	Ametryne	0.9971	Ametryn	0.9926
9	Atrazine	0.9997	Aminocarb	0.9975
10	Benfluralin	0.9988	Amisulbrom	0.9964
11	Benthiavalicarb Isopropyl	0.9981	Ancymidol	0.9967
12	α-BHC	0.9966	Atrazine	0.9965
13	β-BHC	0.9984	Azaconazole	0.9978
14	γ-BHC	0.9978	Azamethiphos	0.9965
15	δ-BHC	0.9997	Azimsulfuron	0.9958
16	Bifenthrin	0.9983	Azinphos-methyl	0.998
17	Biphenyl	0.9963	Azoxystrobin	0.9964
18	Bixafen	0.9993	Beflubutamid	0.9891
19	Boscalid (Nicobifen)	0.9985	Benalaxyl	0.9965
20	Bromophos-Methyl (Bromophos)	0.9966	Bendiocarb	0.9955
21	Bromopropylate	0.9964	Benfuracarb	0.9958
22	Bromuconazol	0.9955	Benodanil	0.9964
23	Bupirimate	0.9972	Benoxacor	0.9973
24	Buprofezin	0.9959	Bensulfuron-methyl	0.9987
25	Cadusafos	0.9959	Benthiavalicarb-isopropyl	0.9973
26	Carboxin	0.9981	Benzoximate	0.9878
27	Chinomethionat	0.9959	Benzoylprop-Ethyl	0.9923
28	Chlorbenside	0.9969	Bifenazate	0.9894
29	Chlorfenapyr	0.9989	Bifenox	0.9953
30	Chlorfenvinphos	0.9969	Bitertanol	0.991
31	Chlorobenzilate	0.996	Boscalid	0.9973
32	cis-Chlordane	0.9981	Brodifacoum	0.9973
33	trans-Chlordane	0.9975	Bromophos-methyl	0.9924
34	Chloropropylate	0.9975	Bromuconazole	0.9915
35	Chlorothalonil	0.9967	Bupirimate	0.9948
36	Chlorpropham	0.9977	Buprofezin	0.9946
37	Chlorpyrifos-ethyl	0.9963	Butachlor	0.9981
38	Chlorpyrifos-methyl	0.9963	Butafenacil	0.9891
39	Chlorthal-Dimethyl (Dacthal)	0.9992	Butoxycarboxim	0.9999
40	Chlorthion	0.9976	Cadusafos	0.9994

41	Cyanazine	0.9978	Carbaryl	0.9968
42	Cyfluthrin (sum of isomers)	0.9981	Carbendazim	0.9973
43	Cyhalofop Butyl	0.9914	Carbetamide	0.9953
44	Cyhalothrin I (λ)	0.9987	Carbofuran	0.9969
45	Cypermethrin (sum of isomers)	0.9972	Carbofuran-3-hydroxy	0.9958
46	Cyprazine	0.9926	Carfentrazone-ethyl	0.9915
47	Cyproconazole	0.9951	Carpropamid	0.9957
48	Cyprodinil	0.992	Chlorantraniliprole	0.9973
49	DDD p,p	0.9926	Chlorbromuron	0.9932
50	DDE p,p	0.9996	Chlorfenvinphos	0.9926
51	DDT p,p	0.9967	Chlorfluazuron	0.998
52	Deltamethrin	0.9968	Chloridazon	0.9976
53	Demeton-S-methyl	0.9917	Chlorotoluron	0.9971
54	Diazinon	0.9912	Chloroxuron	0.9949
55	Dichlobenil	0.9949	Chlorpyrifos	0.998
56	Dichlofluanid	0.998	Cinosulfuron	0.9967
57	4,4-Dichlorobenzophenone	0.9952	Clethodim	0.9942
58	Dichlorvos	0.9967	Clofentezine	0.9966
59	Diclobutrazol	0.9952	Clomazone	0.9935
60	Dicloran (Bortran)	0.9932	Clothianidin	0.9948
61	Dicofol	0.9952	Coumaphos	0.9854
62	Dieldrin	0.997	Crotoxyphos	0.9956
63	Difenoconazole	0.9957	Cyanazine	0.9974
64	Dimethoate	0.9975	Cyazofamid	0.9984
65	Dimethomorph	0.9976	Cycloate	0.9975
66	Disulfoton	0.9992	Cycloheximide	0.9975
67	Diphenylamine	0.9924	Cycloxydim	0.9972
68	Endrin	0.9994	Cycluron	0.9968
69	Endrin aldehyde	0.9979	Cyflufenamid	0.9937
70	Endrin ketone	0.9982	Cymoxanil	0.9938
71	α-Endosulfan	0.9972	Cyproconazole	0.9967
72	β-Endosulfan	0.995	Demeton-S-methyl	0.9913
73	Endosulfan Sulfate	0.9943	Demeton-S-methyl sulfone	0.9963
74	Epn	0.9953	Desmedipham	0.9971
75	Epoxiconazole	0.9973	Desmethyl-pirimicarb	0.9979
76	Eptc	0.9945	Desmetryne	0.9958
77	Ethion	0.9979	Diallate	0.9964
78	Ethoprop (Ethoprophos)	0.9965	Dichlormid	0.9944
79	Etofenprox	0.9958	Diclobutrazol	0.9924
80	Etridiazole (Terrazole)	0.9985	Dicrotophos	0.9829
81	Fenamidone	0.9941	Diethofencarb	0.9975
82	Fenamiphos	0.993	Difenoconazole	0.997
83	Fenarimol	0.998	Diflubenzuron	0.9952
84	Fenazaquin	0.9952	Diflufenican	0.9956

85	Fenbuconazol	0.994	Dimefuron	0.9955
86	Fenchlorfos	0.9996	Dimethenamid	0.9974
87	Fenitrothion	0.9973	Dimethoate	0.9955
88	Fenoxycarb	0.9931	Dimethomorph	0.9963
89	Fenpropathrin	0.9959	Dimoxystrobin	0.9911
90	Fenpropidin	0.9977	Diniconazole	0.9958
91	Fenpropimorph	0.9994	Dinotefuran	0.9982
92	Fenthion	0.9971	Dioxacarb	0.9957
93	Fenvalerate	0.9988	Diphenamid	0.9966
94	Fipronil sulfone	0.9966	Disulfoton sulfone	0.9961
95	Flonicamid	0.9927	Disulfoton sulfoxide	0.9968
96	Fludioxonil	0.9988	Dithiopyr	0.9975
97	Flufenacet	0.998	Diuron	0.9949
98	Flumioxazin	0.9908	Dnoc	0.9987
99	Fluquinconazole	0.9954	Dodemorph	0.9969
100	Flurtamone	0.9985	Dodine	0.9978
101	Flusilazole	0.9988	Edifenphos	0.9932
102	Flutriafol	0.9976	Emamectin-B1A-benzoate	0.9979
103	Fonofos	0.9988	Emamectin-B1B-benzoate	0.9979
104	Formothion	0.9972	Epoxiconazole	0.9955
105	Heptachlor	0.9988	Eprinomectin	0.9934
106	Heptachlor epoxide	0.9984	Esprocarb	0.9959
107	Hexaconazole	0.9972	Etaconazole	0.997
108	Indoxacarb	0.9974	Ethiofencarb	0.9885
109	Iprodione	0.9968	Ethiofencarb sulfone	0.9971
110	Isofenphos-methyl	0.998	Ethiofencarb sulfoxide	0.9914
111	Kresoxim-methyl	0.9967	Ethiprole	0.993
112	Malathion	0.9974	Ethirimol	0.9981
113	Mepanipyrim	0.9922	Ethofumesate	0.9969
114	Metalaxyl	0.9974	Ethoprophos(Ethoprop)	0.9983
115	Methoxychlor	0.993	Ethoxyquin	0.9981
116	Methacrifos	0.9963	Etofenprox	0.9987
117	Methidathion	0.9966	Etoxazole	0.9971
118	Metribuzin	0.9941	Famoxadone	0.9951
119	Mevinphos	0.9925	Fenamidone	0.9967
120	Myclobutanil	0.9983	Fenamiphos	0.9934
121	Nitrofen	0.9966	Fenamiphos sulfone	0.9974
122	o-Phenylphenol	0.9976	Fenamiphos sulfoxide	0.9978
123	Oxadiazon	0.9991	Fenarimol	0.9951
124	Oxadixyl	0.997	Fenazaquin	0.9977
125	Oxyfluorfen	0.997	Fenbuconazole	0.985
126	Parathion-ethyl	0.9967	Fenhexamid	0.9956
127	Parathion-methyl	0.9946	Fenobucarb	0.9962
128	Penconazole	0.993	Fenoxanil	0.9949
129	Pendimethalin	0.9969	Fenoxycarb	0.9977

130	Pentachloroanisole	0.9995	Fenpropimorph	0.997
131	Permethrin	0.9988	Fenpyroximate	0.9967
132	Phenthoate	0.9998	Fensulfothion	0.9944
133	Phosalone	0.9963	Fenthion	0.9903
134	Phosmet	0.9944	Fenthion-oxon sulfone	0.9968
135	Phosphamidon	0.9915	Fenthion sulfoxide	0.9959
136	Piperonyl butoxide	0.9973	Fenuron	0.996
137	Pirimicarb	0.9969	Fipronil	0.9961
138	Pirimicarb-p-desmetyl	0.9978	Fipronil sulfone	0.9932
139	Pirimiphos-Ethyl	0.9969	Florasulam	0.9963
140	Procymidone	0.9991	Fluazifop-p-butyl	0.9972
141	Profenofos	0.9988	Fluazinam	0.9955
142	Prometryn	0.9993	Flubendiamide	0.9871
143	Propachlor	0.994	Flufenacet	0.9969
144	Propanil	0.9933	Flufenoxuron	0.9976
145	Propargite	0.9971	Flumetsulam	0.99
146	Propazine	0.9932	Fluometuron	0.9955
147	Propiconazole	0.9971	Fluopicolide	0.994
148	Propyzamide	0.9935	Fluopyram	0.995
149	Prothiofos	0.9934	Fluorochloridon	0.9944
150	Pyraclostrobin	0.9951	Fluoxastrobin	0.993
151	Pyridaben	0.9986	Fluquinconazole	0.987
152	Pyridaphenthion	0.999	Flurtamone	0.9936
153	Pyrimethanil	0.9939	Flusilazole	0.9939
154	Pyriproxyfen	0.9988	Flutolanil	0.9968
155	Quinoxyfen	0.9962	Flutriafol	0.9968
156	Quintozene	0.9929	Fonofos	0.9937
157	Simazine	0.9975	Forchlorfenuron	0.9987
158	Spirodiclofen	0.998	Forchlorfenuron	0.999
159	Spiromesifen	0.9984	Fosthiazate	0.9964
160	τ-Fluvalinate	0.9966	Fuberidazole	0.9969
161	Tebuconazole	0.9919	Furalaxyl	0.9967
162	Tebufenpyrad	0.9982	Furathiocarb	0.9961
163	Tecnazene	0.9977	Griseofulvin	0.9962
164	Tefluthrin	0.9995	Halofenozide	0.9989
165	Tetraconazole	0.9974	Halosulfuron-methyl	0.994
166	Tetradifon	0.996	Haloxyfop-methyl	0.9974
167	Tetrahydrophthalimide (THPI)	0.9922	Heptenophos	0.9971
168	Terbutryn	0.9967	Hexaconazole	0.9928
169	Tolclofos-methyl	0.9981	Hexaflumuron	0.9974
170	Tolyfluanid	0.9969	Hexazinone	0.9965
171	Triadimefon	0.9981	Hexythiazox	0.9957
172	Triallate	0.9988	Hydramethylnon	0.9966
173	Trifloxystrobin	0.998	Imazalil	0.9955
174	Trifluralin	0.9965	Imibenconazole	0.9984

175	Triticonazole	0.9971	Imidacloprid	0.9925
176	Vinclozolin	0.9997	Indoxacarb	0.9979
177			Iodosulfuron-methyl	0.9966
178			Ipconazole	0.9946
179			Iprovalicarb	0.9944
180			Isoprocarb	0.9973
181			Isoprothiolane	0.9958
182			Isoproturon	0.996
183			Isoxaben	0.9973
184			Isoxadifen-ethyl	0.9905
185			Ketoconazole	0.9984
186			Kresoxim-methyl	0.9947
187			Lenacil	0.998
188			Linuron	0.997
189			Lufenuron	0.9952
190			Malaoxon	0.9969
191			Mandipropamid	0.996
192			Mecarbam	0.9897
193			Mefenacet	0.9967
194			Mefenpyr-diethyl	0.9911
195			Mepanipyrim	0.9976
196			Mepronil	0.9943
197			Metaflumizone	0.9964
198			Metamitron	0.9987
199			Metazachlor	0.9962
200			Metconazole	0.9963
201			Methabenzthiazuron	0.9963
202			Methamidophos	0.9977
203			Methiocarb	0.996
204			Methiocarb sulfone	0.996
205			Methiocarb sulfoxide	0.9931
206			Methomyl	0.9985
207			Methoprotryne	0.9966
208			Methoxyfenozide	0.9899
209			Methyl-Paraoxon	0.9935
210			Metobromuron	0.9959
211			Metolachlor	0.9973
212			S-Metolachlor	0.9979
213			Metolcarb	0.9981
214			Metosulam	0.9986
215			Metoxuron	0.9966
216			Metrafenone	0.9956
217			Metsulfuron-methyl	0.998
218			Mevinphos	0.9971
219			Monocrotophos	0.9962

220		Monolinuron	0.9968
221		Myclobutanil	0.9982
222		Napropamide	0.9948
223		Neburon	0.9866
224		Nitenpyram	0.998
225		Novaluron	0.9971
226		Nuarimol	0.9935
227		Ofurace	0.9978
228		Omethoate	0.9918
229		Oxadixyl	0.9975
230		Oxasulfuron	0.9971
231		Oxycarboxin	0.9974
232		Paclobutrazol	0.9967
233		Paraoxon	0.9963
234		Penconazole	0.9975
235		Pencycuron	0.9877
236		Phenmedipham	0.994
237		Phenthoate	0.9997
238		Phorate sulfone	0.993
239		Phorate sulfoxide	0.9931
240		Phosalone	0.997
241		Phosmet	0.9972
242		Phoxim	0.9967
243		Picoxystrobin	0.998
244		Piperonyl butoxide	0.9968
245		Piperophos	0.9973
246		Pirimicarb	0.9959
247		Pirimisulfuron-methyl	0.9972
248		Prochloraz	0.9967
249		Profenophos	0.9942
250		Promecarb	0.9957
251		Prometon	0.9958
252		Prometryn	0.9969
253		Propanil	0.9758
254		Propaquizafop	0.9984
255		Propazine	0.9929
256		Propetamphos	0.9965
257		Propham	0.9988
258		Propiconazole	0.9975
259		Propoxur	0.9964
260		Propyzamide	0.9942
261		Proquinazid	0.9969
262		Prosulfocarb	0.9947
263		Prothioconazole	0.9925
264		Pyracarbolid	0.9905

265		Pyraclostrobin	0.9925
266		Pyrazophos	0.9938
267		Pyridalyl	0.9993
268		Pyrifenox	0.9963
269		Pyrimethanil	0.9986
270		Pyroxsulam	0.9931
271		Quinalphos	0.9957
272		Quinoxyfen	0.998
273		Resmethrin	0.9979
274		Rimsulfuron	0.9947
275		Rotenone	0.9905
276		Saflufenacil	0.9961
277		Secbumeton	0.9958
278		Sethoxydim	0.997
279		Siduron	0.9966
280		Silthiofam	0.9939
281		Simeconazole	0.9954
282		Simetryn	0.9964
283		Spinetoram	0.9974
284		Spinosad-A	0.9965
285		Spinosad-D	0.9973
286		Spiromesifen	0.996
287		Spirotetramat	0.9955
288		Spiroxamine	0.9977
289		Sulfentrazone	0.9966
290		Sulfotep	0.988
291		Tebuconazole	0.9953
292		Tebufenozide	0.9975
293		Tebufenpyrad	0.9937
294		Tebuthiuron	0.9936
295		Teflubenzuron	0.9942
296		Temephos	0.9967
297		Tepraloxydim	0.9946
298		Terbacil	0.9957
299		Terbumeton	0.9984
300		Terbuthylazine	0.9959
301		Terbutryn	0.9966
302		Tetraconazole	0.9988
303		Tetramethrin	0.996
304		Thiabendazole	0.9972
305		Thiacloprid	0.9957
306		Thiamethoxam	0.9957
307		Thidiazuron	0.9978
308		Thifensulfuron-methyl	0.9981
309		Thifluzamide	0.9944

310		Thiobencarb	0.9986
311		Thiodicarb	0.9958
312		Thiophanate-methyl	0.996
313		Tralkoxydim	0.997
314		Triadimefon	0.9987
315		Triadimenol	0.9991
316		Triallate	0.9949
317		Triazophos	0.9986
318		Trichlorfon	0.9965
319		Tricyclazole	0.9984
320		Trietazine	0.9956
321		Trifloxystrobin	0.9956
322		Triflumizole	0.9973
323		Triflumuron	0.995
324		Tritosulfuron	0.994
325		Vamidothion	0.9956
326		Zoxamide	0.9779

Questionnaire - assessment of pastries filled with cherry filling

Please take 10 to 15 minutes of your time, try the 2 provided coded samples of PASTRIES and fill out the following survey.

Before performing each test, please read the instructions carefully and enter the sample codes in the different fields provided in each individual test.

Thank you for participating in the sensory evaluation and filling out the survey!

The implementation of the consumer test - evaluation of pastries filled with cherry filling - was approved by the Ethics Committee of the Scientific Institute for Food Technologies in Novi Sad (No. 175/I/30-2). The data obtained from this consumer test will be processed and published exclusively in the form of a scientific paper. By answering "YES" at the bottom of the page, you consent to the data being used for the stated purposes.

Do you agree to participate in the consumer test under the above conditions?

☐ Yes

☐ No

Demographic data

Check the appropriate box

Gender:

☐ Male

☐ Female

Age:

☐ Below 30

☐ 30 to 45

☐ 45 to 60

☐ Above 60

Test 1

After you have tried both samples, please indicate your liking for the following properties.

Note: Don't forget to enter the corresponding sample code!

Scale:

1 – Unacceptable

2 - Needs improvement

3 - Ok

4 – Acceptable

5 - Extremely acceptable

Sample 1 (CODE _______)

		1	2	3	4	5	
Appearance	*(Unacceptable)*	☐	☐	☐	☐	☐	*(Extremely acceptable)*
Odour	*(Unacceptable)*	☐	☐	☐	☐	☐	*(Extremely acceptable)*
Taste	*(Unacceptable)*	☐	☐	☐	☐	☐	*(Extremely acceptable)*
Aroma	*(Unacceptable)*	☐	☐	☐	☐	☐	*(Extremely acceptable)*
Texture	*(Unacceptable)*	☐	☐	☐	☐	☐	*(Extremely acceptable)*

Sample 2 (CODE _______)

		1	2	3	4	5	
Appearance	*(Unacceptable)*	☐	☐	☐	☐	☐	*(Extremely acceptable)*
Odour	*(Unacceptable)*	☐	☐	☐	☐	☐	*(Extremely acceptable)*
Taste	*(Unacceptable)*	☐	☐	☐	☐	☐	*(Extremely acceptable)*
Aroma	*(Unacceptable)*	☐	☐	☐	☐	☐	*(Extremely acceptable)*
Texture	*(Unacceptable)*	☐	☐	☐	☐	☐	*(Extremely acceptable)*

Test 2

State your opinion on likability by ticking the appropriate box below.

Note: Don't forget to enter the corresponding sample code!

In relation to the quality of the filling!

Sample 1 (CODE ___)	Sample 2 (CODE ___)	Both	Neither
☐	☐	☐	☐

Test 3

Mark the extent to which the offered product would attract you or refuse to buy it.

Note: Don't forget to enter the corresponding sample code!

Sample 1 (CODE ___)	Sample 2 (CODE ___)
☐ I would be extremely attracted	☐ I would be extremely attracted
☐ I would be very attracted	☐ I would be very attracted
☐ I would be moderately attracted	☐ I would be moderately attracted
☐ I would be neither attracted nor repulsed	☐ I would be neither attracted nor repulsed
☐ I would be moderately repulsed	☐ I would be moderately repulsed
☐ I would be very repulsed	☐ I would be very repulsed
☐ I would be extremely repulsed	☐ I would be extremely repulsed

Test 4

Rate which sample you like better by ticking the box below

Note: Don't forget to enter the corresponding sample code!

Sample 1 (CODE ____)	Sample 2 (CODE ____)
☐	☐

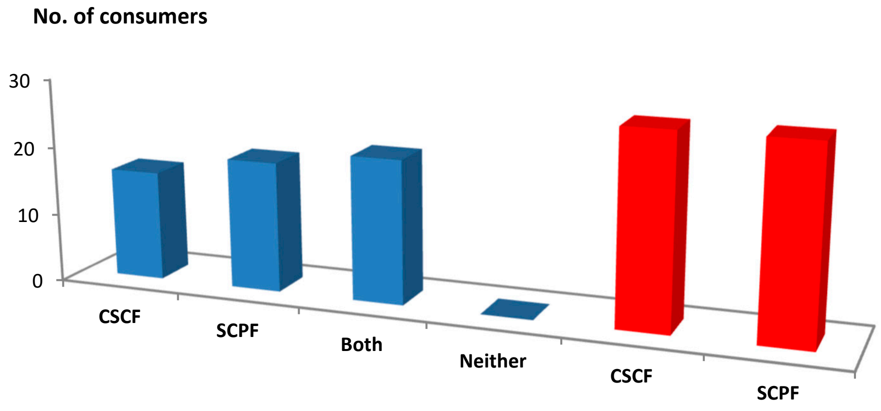

Figure S1. Preference of sour cherry fillings. Test 2 in blue, test 4 in red.

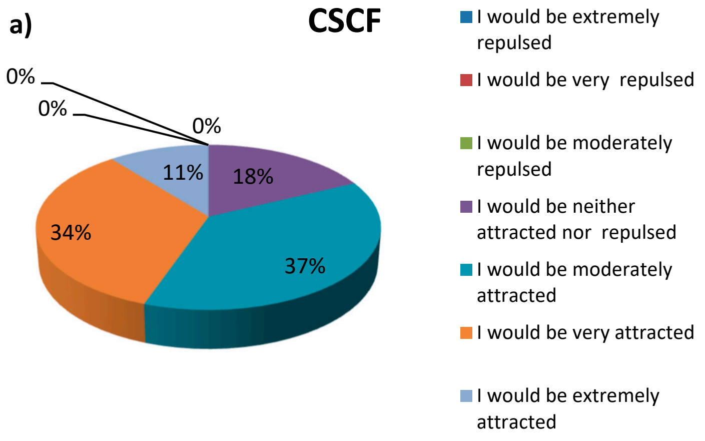

b)

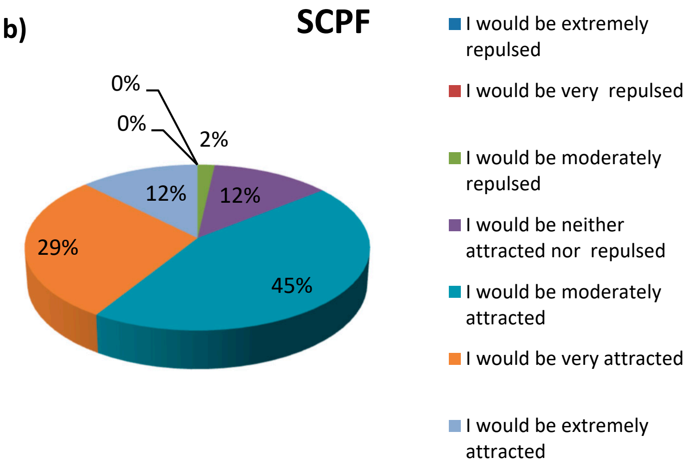

Figure S2. Attractiveness of pastries with a) commercial sour cherry filling b) sour cherry pomace filling
